# Effects of caloric overload before caloric restriction in the murine heart

**DOI:** 10.18632/aging.203967

**Published:** 2022-03-28

**Authors:** Martin Maldonado, Jianying Chen, Huiqin Duan, Shuling Zhou, Lujun Yang, Mazhar Ali Raja, Tianhua Huang, Gu Jiang, Ying Zhong

**Affiliations:** 1 Reproductive Medicine and Genetics, Chengdu Jinjiang Hospital for Maternal and Child Health Care, Chengdu 610066, China; 2 Translational Medical Center, Second Affiliated Hospital of Shantou University Medical College, Shantou, Guangdong 515041, China

**Keywords:** caloric restriction, cardiac function, mitochondria, AMPK, PGC1-α, SIRT1, mTOR, telomerase

## Abstract

The beneficial effects of caloric restriction (CR) against cardiac aging and for prevention of cardiovascular diseases are numerous. However, to our knowledge, there is no scientific evidence about how a high-calorie diet (HCD) background influences the mechanisms underlying CR in whole heart tissue (WHT) in experimental murine models.

In the current study, CR-treated mice with different alimentary backgrounds were subjected to transthoracic echocardiographic measurements. WHT was then analyzed to determine cardiac energetics, telomerase activity, the expression of energy-sensing networks, tissue-specific adiponectin, and cardiac precursor/cardiac stem cell markers.

Animals with a balanced diet consumption before CR presented marked cardiac remodeling with improved ejection fraction (EF) and fractional shortening (FS), enhanced OXPHOS complex I, III, and IV, and CKMT2 enzymatic activity. Mice fed an HCD before CR presented moderate changes in cardiac geometry with diminished EF and FS values, but improved OXPHOS complex IV and CKMT2 activity.

Differences in cardiac remodeling, left ventricular systolic/diastolic performance, and mitochondrial energetics, found in the CR-treated mice with contrasting alimentary backgrounds, were corroborated by inconsistencies in the expression of mitochondrial-biogenesis-related markers and associated regulatory networks. In particular, disruption of eNOS and AMPK -PGC-1α-mTOR-related axes.

The impact of a past habit of caloric overload on the effects of CR in the WHT is a scarcely explored subject that requires deeper study in combination with analyses of other tissues and organs at higher levels of organization within the organ system. Such research will eventually lead to the development of preventative and therapeutic strategies to promote health and longevity.

## INTRODUCTION

Caloric overload, obesity, and aging are associated with cardiovascular disorders and other related pathophysiological and metabolic conditions such as lipotoxicity, disrupted oxidative phosphorylation (OXPHOS), insulin resistance, redox stress, inflammation, cardiac hypertrophy, and diastolic/systolic dysfunction [1–3]. These conditions are exacerbated by aging.

In contrast, caloric restriction (CR) positively affects the physiological and pathophysiological events related to aging and lifespan in several species, including mammals and rodents [4–6].

Preliminary trials in animal models and humans have revealed that CR without malnutrition delays cardiac aging, prevents cardiovascular disease, reduces arterial hypertension and endothelial dysfunction, induces cardioprotection by preserving cardiac contractile and cardiomyocyte function, and lessens cardiac remodeling [7–12].

These mechanisms are orchestrated by a wide range of biochemical and cellular events, involving redox homeostasis, inflammation [13, 14], apoptosis [15], autophagy [16, 17], telomerase activity [18], mitochondrial biogenesis/function [19], energy-sensing pathways [20–26], and adipokines [27], among other components.

The myocardium has an enormous and steady requirement for energy that is generated by fatty acid oxidation (FAO) via OXPHOS, which occurs in the mitochondrial matrix and produces ~95% of the ATP needed to maintain cardiac activities [28]. However, the capacity for mitochondrial oxidative metabolism is tuned at the transcriptional and translational levels. For instance, activation of the energy homeostasis-regulating AMPK -SIRT1- mTOR and PGC-1α-SIRT1-AMPK networks confers cardioprotective benefits and delays diabetes and related metabolic disorders in mammals [26, 27, 29], in part through control of energy expenditure and mitochondrial respiration, biogenesis, and function [26, 30–32].

Mitochondria are chronically exposed to reactive oxygen molecules. Consequently, different tissues, particularly the heart, are subjected to an age-associated increase in reactive oxidant-induced mitochondrial DNA (mtDNA) damage [33]. CR has been suggested to synergize with telomerase expression to result in significant lifespan extension [34], and telomere shortening is associated with aging [35]; thus, telomere dynamics may play a role in the systemic effects of CR.

Perhaps the component most noticeably perturbed by caloric overload or CR is the adipose tissue, which is now recognized as an endocrine organ that secretes multiple peptides that are together referred to as adipokines. Emerging evidence suggests that adipokines play a vital role in the regulation of the cardiovascular system [7]. At the cellular and molecular levels, the adipokine adiponectin (and its receptors), whose expression and serum levels decrease in obesity and increase after weight loss [36, 37], exerts anti-inflammatory, antioxidant, antiapoptotic, and antihypertrophic activity, which is crucial in cardioprotection [38–44].

CR has also been suggested to attenuate the decreases in cardiomyocyte number and function in the aging heart, possibly due to cardiomyocyte turnover from endogenous cardiac stem cells (CSCs), cardiac progenitor cells (CPCs), or both [45–47]. Whether the adult heart possesses CSCs for cardiomyocyte regeneration is an important yet controversial subject in cardiovascular and regenerative medicine [48].

To the best of our knowledge, there is no evidence regarding how the effects of short-term CR in the whole heart tissue (WHT) of mice are impacted by a high-calorie diet (HCD) background.

Based on the aforementioned beneficial effects of CR in the heart and cardiovascular system and our most recent findings suggesting that caloric overload prior to CR exerts (to some degree) positive effects on skeletal muscle function and energy metabolism [49], the present work aimed to investigate the impacts of past habits of caloric overload on some of the most relevant mechanisms and molecular pathways that interact to modulate CR-elicited genetic, metabolic, and functional changes in the WHT in a murine model.

## MATERIALS AND METHODS

### Food design

The animal food was developed by Beijing Keao Xieli Feed Co., Ltd. (Beijing Chaoyang District, Yangshan Road, Number 4). The standard food (3.1 kcal/kg) formulation supplied by the manufacturer is shown in Supplementary Table 1. The HCD food was developed by proportionally increasing the macronutrients (total lipids and carbohydrates) in the standard food to achieve 5.5 kcal/kg. The HCD was a hypercaloric formula designed to induce obesity and metabolic disorders in the experimental animal model (Supplementary Table 2).

### Generation of the animal model and dietary interventions

Female CD-1 (ICR) mice were maintained in a specific pathogen-free animal facility in individually ventilated cages and were housed at 23°C under a 12-hour dark/light cycle. Water and food were given *ad libitum* prior to the dietary interventions. All animal work was conducted using protocols approved by the Institutional Review Board of the Chengdu Jinjiang Maternity and Child Health Hospital.

**Figure 1:**
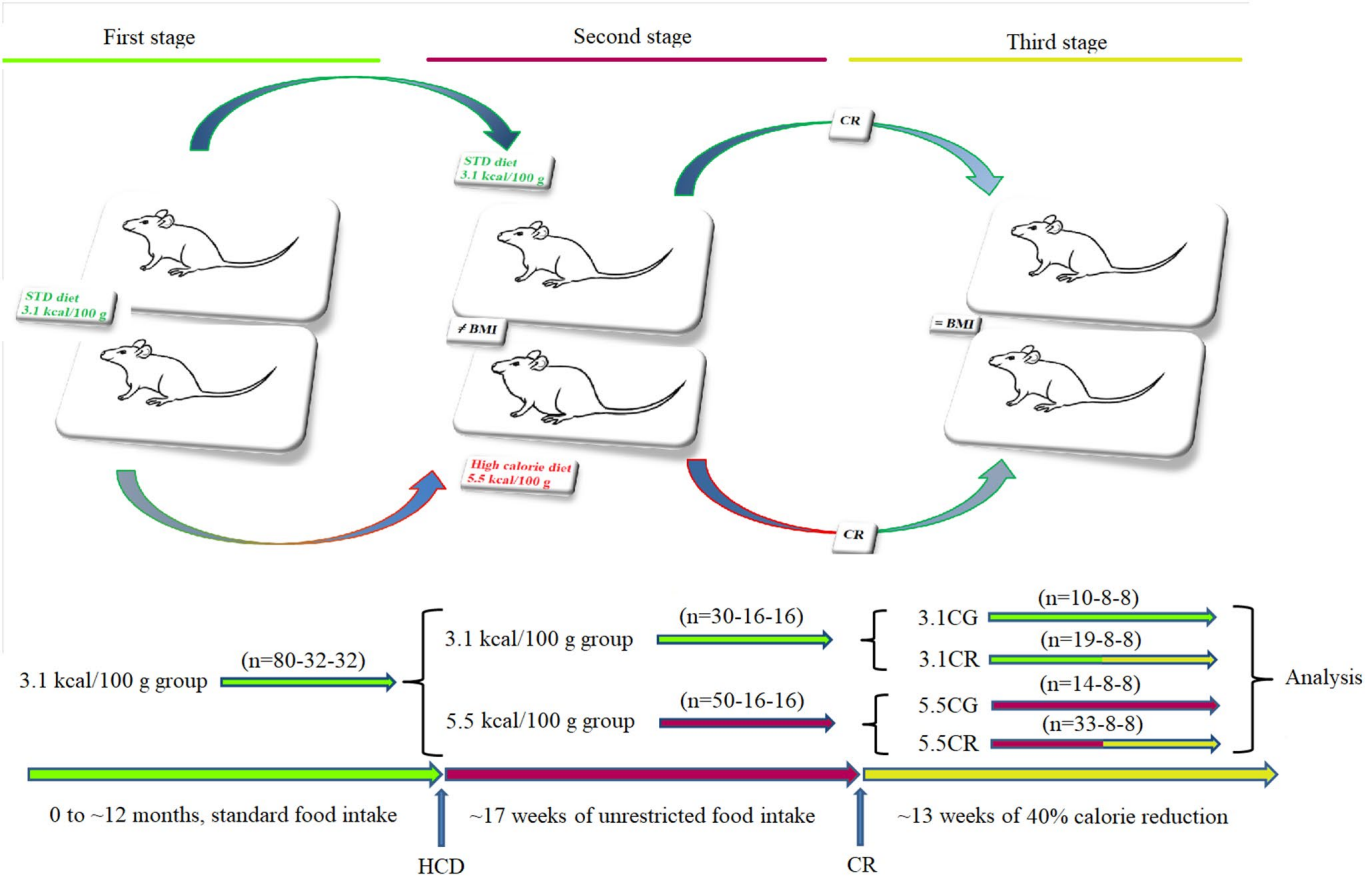
**Generation of the experimental animals.** Representative graphics for the characterization of the animal models. CD-1 mice fed for 12 months with standard food *ad libitum* (first stage) were separated into animals fed standard food *ad libitum* and animals fed an HCD *ad libitum* for a period of 17 weeks (second stage). Some animals were subjected to CR for a period of 13 weeks before sacrifice (third stage). The experimental groups included mice fed a 3.1 kcal/kg diet and subjected to CR (the 3.1CR group) and animals with an HCD background subjected to CR (the 5.5CR group).

For generation of the experimental models (Figure 1), three cohorts of animals were developed: one cohort for measurement of metabolic and genetic variables (*n* = 80), one cohort for measurement of lipid content by acid hydrolysis assay (*n* = 32), and one cohort for determination of cardiac function/geometry and mitochondrial energetics (*n* = 32).

Briefly, ~12-month-old mice (*n* = 80-32-32) fed 3.1 kcal/kg standard food *ad libitum* (first stage) were divided (second stage) into two groups: a group fed standard food *ad libitum* (*n* = 30-16-16) and another group fed a 5.5 kcal/kg HCD *ad libitum* for 17 weeks (*n* = 50-16-16). The mice initially fed 3.1 kcal/kg standard food that continued with the same diet (*n* = 10-8-8) became the 3.1 kcal/kg control group (3.1CG). The animals initially fed standard food that were then subjected to CR for 13 weeks with a low-calorie diet consisting of 60% of the *ad libitum* 3.1 kcal/kg calorie intake (*n* = 19-8-8), became the 3.1CR group (3.1CR). The animals fed the 5.5 kcal/kg HCD diet *ad libitum* for 17 weeks that continued with the same diet (*n* = 14-8-8) became the 5.5 kcal/kg control group (5.5CG). The animals fed the 5.5 kcal/kg HCD *ad libitum* for 17 weeks that were switched to “standard food” with 60% of the calories in the HCD and fed *ad libitum* for 13 weeks (*n* = 33-8-8) became the 5.5CR group (5.5CR). The daily amount of food of the CR-treated animals was periodically adjusted according to changes in body weight to maintain nutritional balance relative to the body mass index (BMI). The transition from the standard food or HCD *ad libitum* to CR was gradually achieved over a period of one week.

The mice were monitored daily by laboratory members and animal health technicians. Before the experimental endpoint, the mice experienced minimal pain and stress during routine handling, body weight determination, echocardiography procedures, and blood collection from the tail vein to measure blood glucose levels.

No ill or deceased mice were observed before the experimental endpoint (when the mice were 19 to 20 months old). The animals were euthanized by the cervical dislocation technique.

### Transthoracic echocardiographic measurement

Cardiac function and geometry were evaluated in anesthetized (1.5% isoflurane) mice using a 2-D guided M-mode Vevo-3100 echocardiograph (FUJIFILM, Japan) equipped with a 9- to 70-MHz UHF probe. The transmission frequency was set at 30 MHz, the acquisition gain was 27.0 dB, and the dynamic range was 60 dB. The animals were placed on a homeothermic table, and the core temperature was maintained at 37°C. The hearts were imaged in the parasternal long-axis (PLAX) view to assess left ventricular (LV) dimensions and systolic/diastolic parameters. The heart rates in the experimental groups were consistently monitored between ~400 and ~500 bpm. The interventricular septal thickness at diastole (IVS-d), interventricular septal thickness at systole (IVS-s), LV internal end-diastolic diameter (LVIDD), LV internal end-systolic diameter (LVIDs), LV posterior wall dimension-diastole (LVPW-d), and LV posterior wall dimension-systole (LVPW-s) were measured. The ejection fraction (EF; %), fractional shortening (FS; %), LV mass (mg), corrected LV mass (mg), LV end-diastolic volume (LVvol-d; μL), and LV end-systolic volume (LVvol-s; μL) were automatically calculated with FUJIFILM VisualSonics, Inc. Vevo LAB 3.1.0 image analysis software. All measures derived from echocardiography were obtained by averaging the readings of three consecutive and complete cardiac cycles.

### Tissue collection

WHT was collected and weighed after completely removing the blood with ice-cold PBS. Samples were immediately analyzed or flash-frozen in liquid nitrogen and stored until execution of the experiments.

### Acid hydrolysis assay for determination of the total lipid content in WHT

The acid hydrolysis method was performed according to the National Standard of the People’s Republic of China GB 5009.6-2016. Briefly, the WHT samples (*n* = 8 per group) were placed into a 25-mL conical flask, and 5 mL of 2 mol/L hydrochloric acid solution was added. The samples were heated on a boiling electric plate for 1 hour and were rotated and shaken once every 10 min. Fifteen milliliters of hot water was added and mixed well, and the mixture was filtered. The precipitate was washed with hot water, and after neutralization, dried in an oven at 100°C ± 5°C for 1 hour and then allowed to cool.

The total fat content in the test sample was calculated as follows:


$$\text{X}=\frac{{\text{m}}_{1}-{\text{m}}_{0}}{{\text{m}}_{2}}\times \text{100}$$

X: weight of fat in the test sample, g/100 g
m_1_: weight of the flask and fat after constant weight, g
m_0_: weight of the flask, g
m_2_: weight of the test sample, g
100: conversion coefficient.

### Mitochondrial OXPHOS complex enzymatic activity assay

NADH-coenzyme Q oxidoreductase (complex I), Q-cytochrome c oxidoreductase (complex III), and cytochrome c oxidase (complex IV) activity levels were assayed with CheKine™ Mitochondrial Complex I, III, and IV Activity Colorimetric Assay Kits (Cat #: KTB1850, Cat #: KTB1870, Cat #: KTB1880; Abbkine, Inc.) according to the instructions provided by the manufacturer.

Briefly, 100 mg of tissue was homogenized on ice with 1 ml of Reagent I and 10 μL of Reagent III. The homogenate was centrifuged at 600 × g for 5 min (4°C), and the supernatant was transferred into a new centrifuge tube and centrifuged at 11,000 × g for 10 min at 4°C. The pellet, which contained the mitochondrial extract, was mixed with 200 μL of Reagent II and 2 μL of Reagent III. Then, the samples were resuspended and used to detect the activity of mitochondrial respiratory chain complexes with a Multiskan GO microplate reader (Thermo Fisher Scientific, Oy Ratastie 2, P.O. Box 100 FI-01621, Finland).

For complex I, 10 μL of the sample, 15 μL of Working Reagent VI, and 200 μL of Working Solution were mixed and poured into a 96-well UV microplate. The absorbance was immediately read at 340 nm (A_1_) and after 2 min (A_2_).

For complex III, 10 μL of the sample, 25 μL of Reagent VI, and 200 μL of working solution were mixed and poured into a 96-well microplate. The absorbance was immediately read at 550 nm (A_1_) and after 2 min (A_2_).

For complex IV, 10 μL of sample and 200 μL of working solution were mixed and poured into a 96-well microplate. The absorbance was immediately read at 550 nm (A_1_) and after 2 min (A_2_). The change in absorbance for each complex enzymatic assay was calculated as follows: ΔA= A_1_–A_2_ (complexes I and IV) or ΔA= A_2_–A_1_ (complex III).

The activity of complexes I, III, and IV was calculated as follows:

In the mitochondrial pellet: (U/g fresh weight) = [ΔA × V Total ÷ (ε × d) × 10^9^] ÷ (W ÷ V Resuspended × V Sample) ÷ T = 731 (complex I), 249 (complex III), or 444 (complex IV) × ΔA ÷ W

Definitions:
U: enzyme activity unit equivalent to the consumption of 1 nmol of NADH (complex I) or reduced cytochrome C (complex III, IV) in 1 g of tissue/min in the reaction system
V Total: total reaction volume (2.1 × 10^4^ L)
ε: reduced cytochrome C molar extinction coefficient (19.1 × 10^3^ mol/L/cm)
d: 96-well plate diameter (0.5 cm)
10^9^: unit conversion factor (1 mol = 10^9^ nmol)
V Sample: sample volume added (0.01 mL)
T: reaction time (1 min)
W: sample weight (g)
ΔA_2_: determination value of pellet
V Resuspended: volume of the resuspended pellet (0.202 mL).

### Mitochondrial creatine kinase (mtCK) s-type (CKMT2) quantitative determination

mtCK, a sarcomeric isoenzyme, was analyzed in the WHT with the Mouse CKMT2 ELISA Kit (Quanzhou Ruixin Biological Technology Co., Ltd.) according to the instruction manual.

Briefly, 50 μL of standard or sample was added to each appropriate well (all standards and samples were added in triplicate). One hundred microliters of enzyme conjugate was added to each standard or sample well except for the blank wells. The plate was covered with an adhesive strip and incubated for 60 min at 37°C. Then, the plate was washed four times. After the washing procedure, all wells were aspirated, and the plate was rewashed four times using Wash Buffer (1X). After the final wash, the plate was inverted and dried by tapping the plate on absorbent paper until no moisture was evident. Substrate A (50 μL) and Substrate B (50 μL) were added to each well and gently mixed, and the plate was incubated for 15 min at 37°C in the absence of light. Then, 50 μL of stop solution was added to each well. Finally, the optical density (OD) was read at 450 nm using a microtiter plate reader within 15 min.

### MtDNA copy number assay

DNA was isolated from WHT of the 3.1CG (*n* = 9), 3.1CR (*n* = 16), 5.5CG (*n* = 14), and 5.5CR (*n* = 30) groups with a TIANamp Genomic DNA Kit (TIANGEN), and the mtDNA was analyzed with a Mouse Mitochondrial DNA Copy Number Assay Kit (Detroit R&D, Inc.) through comparison of mtDNA and nuclear (*n*) DNA measured by quantitative real-time polymerase chain reaction (qPCR).

The mtDNA copy number was calculated as follows:

ΔCt1 = Ct (control mtDNA) − Ct (control nuclear DNA)
ΔCt2 = Ct (experimental group mtDNA) − Ct (experimental group nuclear DNA)

The Mouse Mitochondrial DNA Copy Number Assay Kit was used according to the manufacturer’s manual.

### qPCR assay

Tissues preserved in liquid nitrogen were homogenized, and the RNA was extracted with Takara RNAiso PLUS Total RNA Extraction Reagent (Takara Bio, Inc.). DNA was extracted with a TIANamp Genomic DNA Kit (TIANGEN). The total nucleic acid concentration and OD were assayed by UV spectrophotometry. cDNA was obtained with a Takara kit (RR047Q). qPCR was performed with 100 ng of target DNA. SYBR Premix Ex Taq™ (Takara RR420Q) and an ABI7500 instrument (Applied Biosystems, Foster City, CA, USA) were used to quantify the relative expression of Cluster of Differentiation 29 (CD29); CD34 (hematopoietic progenitor cell antigen); Connexin-37; Connexin-40; GATA binding protein 4 (GATA4); GATA6; ISL1; MyoD; Myf5; Myosin Heavy Chain 6, Alpha Isoform (Myh6); Myosin Heavy Chain 7, Beta Isoform (Myh7); SSEA-1; T-Box Transcription Factor 18 (TBX18); Wilms' tumor suppressor gene (Wt1); Bone Morphogenetic Protein 4 (BMP4); Proto-Oncogene C-KIT (C-KIT); Desmin; NKX2.5; endothelial nitric oxide synthase (eNOS); mammalian Target Of Rapamycin (mTOR); Adiponectin; AdipoR1; T-cadherin; GAPDH (reference gene); and 18S rRNA (reference gene) (Supplementary Table 3).

### Western blot analysis

Tissues were homogenized with 5 volumes of radioimmunoprecipitation assay (RIPA) buffer (Solarbio^®^ Life Sciences), and the supernatants were fractionated by SDS–PAGE. The proteins were quantified with a BCA protein quantification kit (Solarbio^®^ Life Sciences), transferred to PVDF membranes (Bio–Rad), and blocked with 5% blocking solution for Western blotting (Roche). The membranes were then exposed to an anti-eNOS antibody (EPR19296) (ab199956, Abcam; dilution: 1/1000), an anti-AMPK alpha 1 + AMPK alpha 2 antibody (EPR19549) (ab207442, Abcam; dilution: 1/1000), a recombinant anti-AMPK alpha 1 (phospho-T183) + AMPK alpha 2 (phospho-T172) antibody (EPR5683) (ab133448, Abcam; dilution: 1/5000); an anti-PGC1 alpha rabbit pAb (#A11971, ABclonal; dilution: 1/1000), an anti-mTOR (phospho-S2448) antibody (EPR426[2]) (ab109268, Abcam; dilution: 1/5000), an anti-mTOR (phospho-S2481) antibody (EPR427[N]) (ab137133, Abcam; dilution: 1/5000), an anti-adiponectin antibody (EPR17019) (ab181281, Abcam; dilution: 1/1000), and an anti-GAPDH antibody (EPR16891) loading control (ab181602, Abcam; dilution: 1/5000). Immunodetection was conducted using a goat anti-rabbit IgG H&L (HRP) (ab6721, Abcam; dilution: 1/3000) secondary antibody and an enhanced chemiluminescence device (Bio–Rad gel imager).

### NAD-dependent deacetylase Sirtuin-1 assay

One hundred milligrams of WHT homogenate from the 3.1CG (*n* = 10), 3.1CR (*n* = 19), 5.5CG (*n* = 14), and 5.5CR (*n* = 33) groups was analyzed with a Mouse NAD-Dependent Deacetylase Sirtuin-1 (SIRT1) ELISA Kit (CUSABIO) for quantitative determination of SIRT1 according to the manufacturer’s instructions.

### Telomerase detection assay

Homogenates were analyzed with a TRAPeze RT Telomerase Detection Kit (Millipore) for fluorometric detection and real-time quantification of telomerase activity.

Briefly, 50 to 100 mg of frozen WHT was homogenized and resuspended in 200 μl of CHAPS lysis buffer. Samples were incubated on ice for 30 min and centrifuged at 12,000 × g for 20 min (4°C). One hundred sixty microliters of the supernatant was used to determine the protein concentration, and the remaining extract was used to perform the real-time telomerase assay according to the manufacturer’s manual.

### Statistical analysis

The data were analyzed with two-tailed unpaired *t* tests with Welch’s correction for comparisons of two groups; two-way ANOVA with Tukey’s multiple comparison test for the acid hydroxylase assay, echocardiography, and mitochondrial energetics data; and two-way ANOVA with Bonferroni post hoc analysis for determination of the interactions among multiple groups with different sample sizes. Correlations were tested by Pearson analysis, and the data were processed using GraphPad Prism 8.3.0. Western blot analysis and protein densitometry were conducted with Image Lab 5.0. A *P* value < 0.05 was considered to indicate statistical significance.

### Institutional review board statement

The study was conducted according to the guidelines of the Declaration of Helsinki and approved by the Institutional Review Board of Chengdu Jinjiang Hospital for Maternal and Child Health Care. Mice were daily monitored by laboratory members and by animal health technicians.

### Data availability

All data generated or analyzed during this study are included in this published article.

## RESULTS

### Physiological changes during and after the development of the experimental animal model

**Figure 2:**
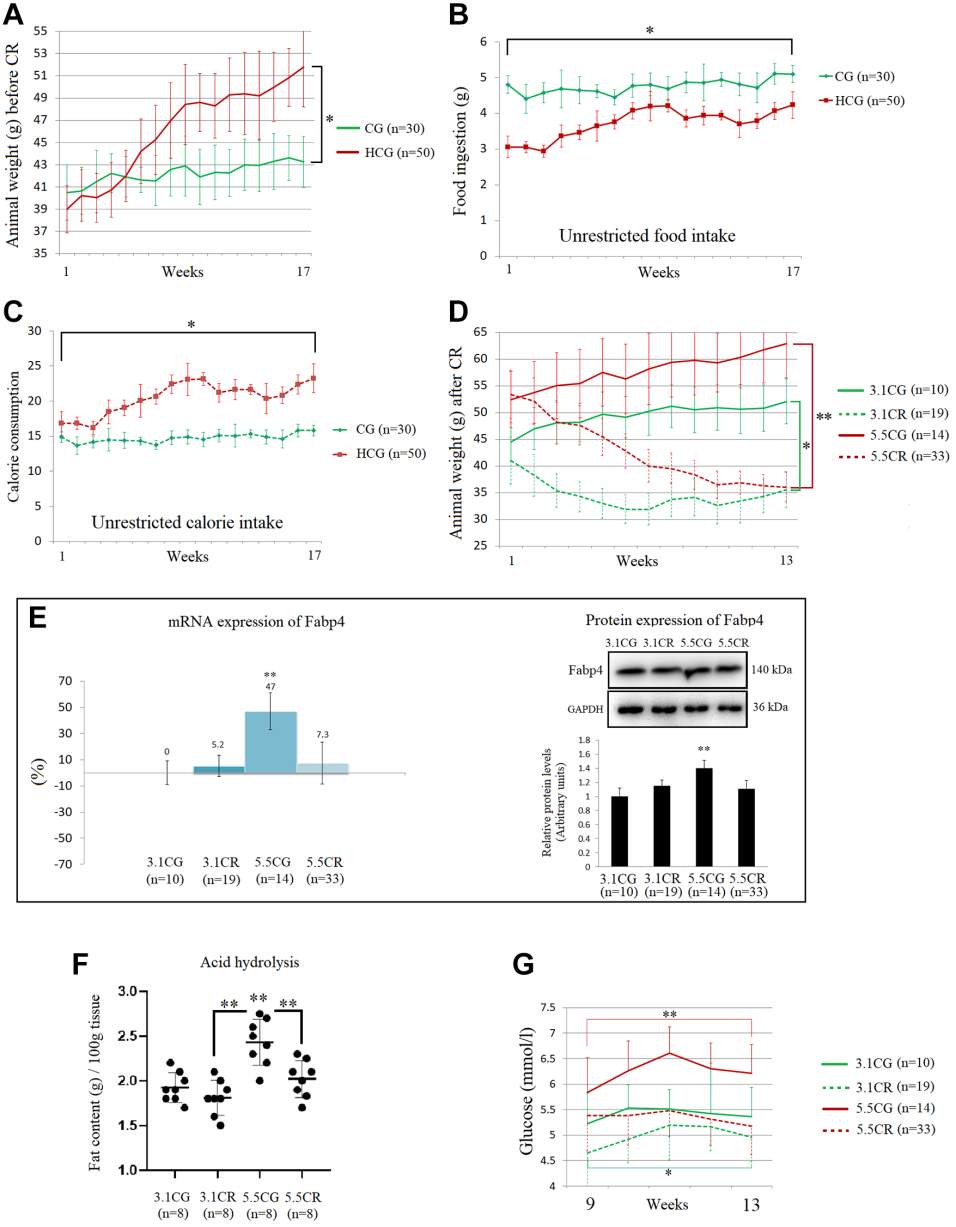
**Physiological changes during and after the development of the experimental animal model.** The graphics are representations of 1 of the 3 cohorts of animals. Average body weights of the animal groups before CR (**A**). Average food ingestion of the animal groups before CR, expressed in grams (**B**). Average caloric intake of the animal groups before CR (**C**). Animal weights during the CR period, expressed in grams (**D**). The mRNA and protein levels of Fabp4 were used to predict INTM and IM fat infiltration (**E**). Fabp4 analyzed by qPCR (mRNA), expressed as %; the 3.1CG value was set to 0, and the compared samples were normalized to this level. Positive % values represent upregulation. Negative values represent downregulation. The protein expression of Fabp4 was obtained by Western blot analysis and quantified with Image Lab 6.1 software. The values were normalized to GAPDH expression; the 3.1CG expression was set to 1.0. Total fat content in the WHT, as determined by acid hydrolysis (**F**). The values are expressed in g/100 g of tissue. Comparison of the blood glucose levels of mice during weeks 9 to 13 of dietary restriction (**G**). The data are the mean ± SD. ^*^P < 0.05; ^**^P < 0.01 vs. the 3.1CG group, unless otherwise specified.

At the end of the second stage of animal model development, mice fed the HCD *ad libitum* (in the high-calorie groups, HCGs) showed significant increases in body weight (Figure 2A). Interestingly, the HCG mice presented lower daily volumes of ingested food (Figure 2B) than the mice fed the standard 3.1 kcal/kg diet *ad libitum* (control group [CG] mice). Nevertheless, due to the food composition, the caloric intake of the HCGs was considerably higher than that of their control counterparts (Figure 2C).

Although the groups with different feeding regimens (3.1 kcal/kg and 5.5 kcal/kg diets *ad libitum*) showed significant disparities in weight at the beginning of CR period, at the end of the CR period, the weights of the mice in the CR-treated (3.1CR and 5.5CR) groups had decreased such that there was no significant difference between them (Figure 2D), and the values in these groups were also significantly lower than those in their control counterparts (the 3.1CG and 5.5CG groups).

The mRNA and protein expression of the adipose-type cytoplasmic fatty acid-binding protein Fabp4 (Figure 2E) was used in this work to predict intermyocellular (INTM) and intramyocellular (IM) fat infiltration [50]. The transcriptional and translational data suggested higher amounts of INTM and IM lipid content in the 5.5CG group than in the other groups analyzed. Moreover, the 3.1CG, 3.1CR, and 5.5CR groups showed no significant differences at the end of the CR period.

Due to the small sizes of mice, it is impossible to distinguish INTM and IM adiposity; thus, all available studies on IM adipogenesis in mice refer to both INTM and IM fat [51]. Moreover, due to the intricate and nonhomogeneous structure of the heart, we analyzed the total lipid content in the heart by the acid hydrolysis technique (Figure 2F). Since this assay requires the use of whole organ tissue, a separate cohort of 8 animals per group was selected according to the experimental animal model standards. WHT was collected and then hydrolyzed with hydrophobic acid, as explained in the Materials and Methods (section 2.5). The results showed a significant increase in the total lipid content in the 5.5CG group, while the rest of the groups presented similar fat content.

In addition to the other variables described above, we included glucose levels in our characterization of the experimental animal model (Figure 2G). As expected, mice subjected to CR showed better blood glucose levels than *ad libitum*-fed mice, while the 5.5CG mice presented the highest values after 8 hours of starvation.

### Cardiac geometry and function

**Figure 3:**
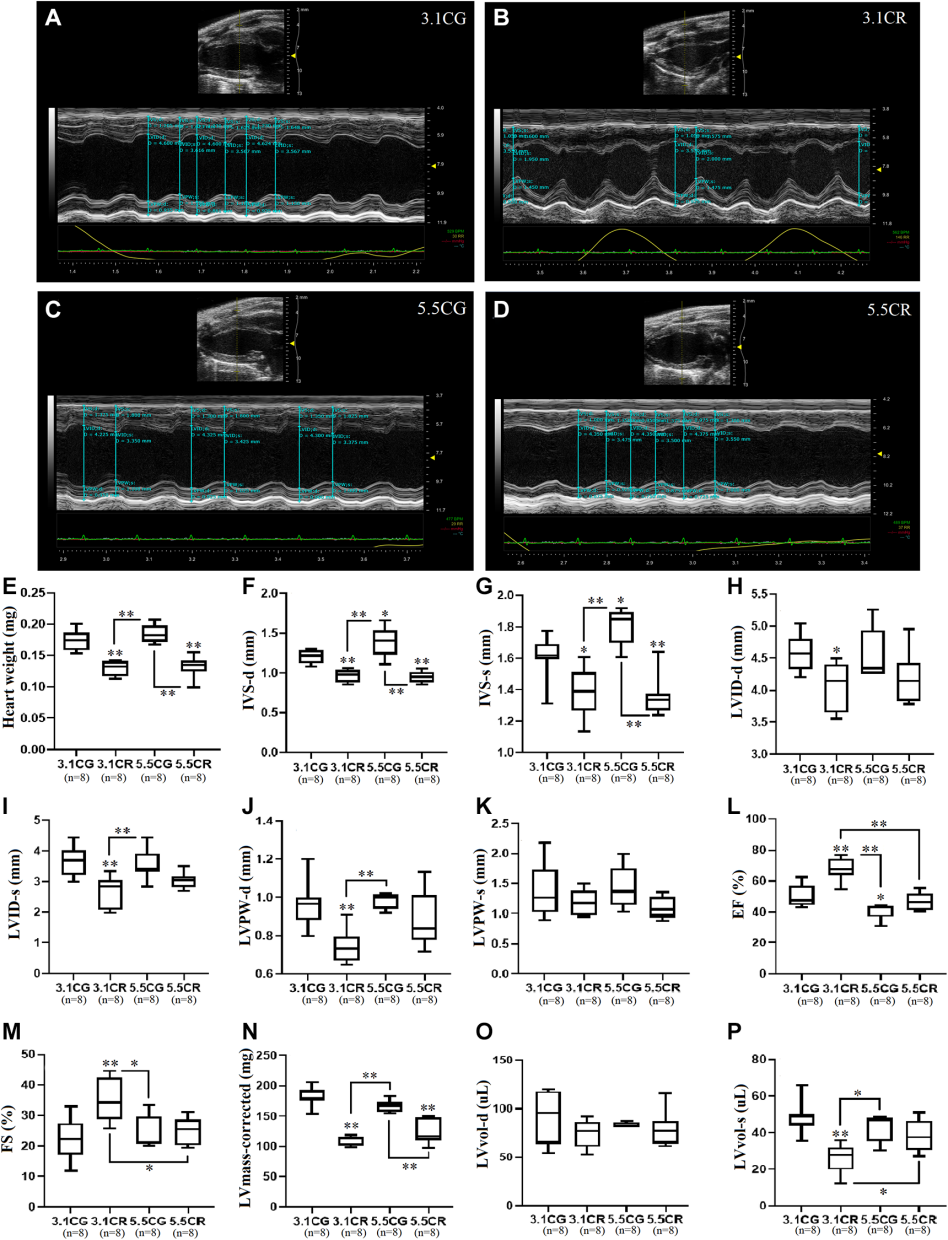
**Transthoracic echocardiographic measurement.** Representative M-mode echocardiographic images and measurements acquired with the FUJIFILM VEVO 3100 imaging system. Images were acquired in the LV PLAX view for the 3.1CG (**A**), 3.1CR (**B**), 5.5CG (**C**), and 5.5CR (**D**) groups. Vevo LAB 3.1.0 image analysis software was used to automatically calculate the weight of the hearts of mice, expressed in mg (**E**); IVS-d, expressed in mm (**F**); IVS-s, expressed in mm (**G**); LVID-d, expressed in mm (**H**); LVID-s, expressed in mm (**I**); LVPW-d, expressed in mm (**J**); LVPW-s, expressed in mm (**K**); EF, expressed in % (**L**); FS, expressed in % (**M**); corrected LV mass, expressed in mg (**N**); LVvol-d, expressed in μl (**O**); and LVvol-s, expressed in μl (**P**). The data are the mean ± SD. ^*^P < 0.05 and ^**^P < 0.01 vs. the 3.1CG group, unless otherwise specified. After echocardiographic measurements, mice were sacrificed by the cervical dislocation technique. The hearts were collected and weighed after complete removal of blood via washing with excess ice-cold PBS.

Representative M-mode echocardiographic images and measurements were obtained in the PLAX view from the left ventricles of 3.1CG (Figure 3A), 3.1CR (Figure 3B), 5.5CG (Figure 3C), and 5.5CR (Figure 3D) mice.

The hearts of CR-treated mice were significantly lighter than those of mice in the CGs (Figure 3E). Echocardiographic analysis revealed that CR in mice previously fed standard food (3.1CR mice) significantly altered cardiac geometry, as evidenced by decreased IVS-d (Figure 3F), IVS-s (Figure 3G), LVID-d (Figure 3H), LVID-s (Figure 3I), and LVPW-d (Figure 3J) values. LVPW-s values showed no significant differences between groups (Figure 3K). LV systolic performance and muscle contractility, as estimated by EF (Figure 3L) and FS (Figure 3M), were markedly improved, while the corrected LV mass (Figure 3N), which was consistent with reductions in gross heart weight (Figure 3E) and LVvol-s (Figure 3P) values, was significantly decreased (vs. 3.1CG group). LVvol-d values showed no significant differences between groups (Figure 3O).

Mice fed an HCD before CR (5.5CR mice) also underwent cardiac remodeling, as verified by decreased IVS-d (Figure 3F) and IVS-s (Figure 3G) values. However, LVID-d (Figure 3H) and LVID-s (Figure 3I) measurements, which also seemed reduced, were not significantly altered. EF (Figure 3L) and FS (Figure 3M) outcomes were significantly worse in the 5.5CR group than in the 3.1CR group. However, these results did not differ among the other groups. As seen in the 3.1CR group, the corrected LV mass (Figure 3N) was significantly decreased in the 5.5CR group, consistent with a change in the gross heart weight (vs. 3.1CG and 5.5CG groups; Figure 3E). LVvol-s (Figure 3P), another clinical parameter reflecting global LV systolic performance and remodeling, was higher in the 5.5CR group than in the 3.1CR group, which may indicate impairment in the contractile tissue.

Animals fed only an HCD (5.5CG mice) displayed heavier bodyweights, but the apparent increase in gross heart weight in the 5.5CG group compared with the 3.1CG group was not significant. Additionally, 5.5CG mice presented increased IVS-d and IVS-s values (Figure 3F and 3G), indicative of LV hypertrophy. Although EF (Figure 3L) analysis revealed poor LV performance and muscle contractility (vs. 3.1CG), no signs of diminished FS (FS strongly correlates with EF under normal ventricular geometry, without regional wall motion abnormalities; Figure 3M) were evidenced.

### Mitochondrial energetics, mtDNA copy numbers, and eNOS expression

**Figure 4:**
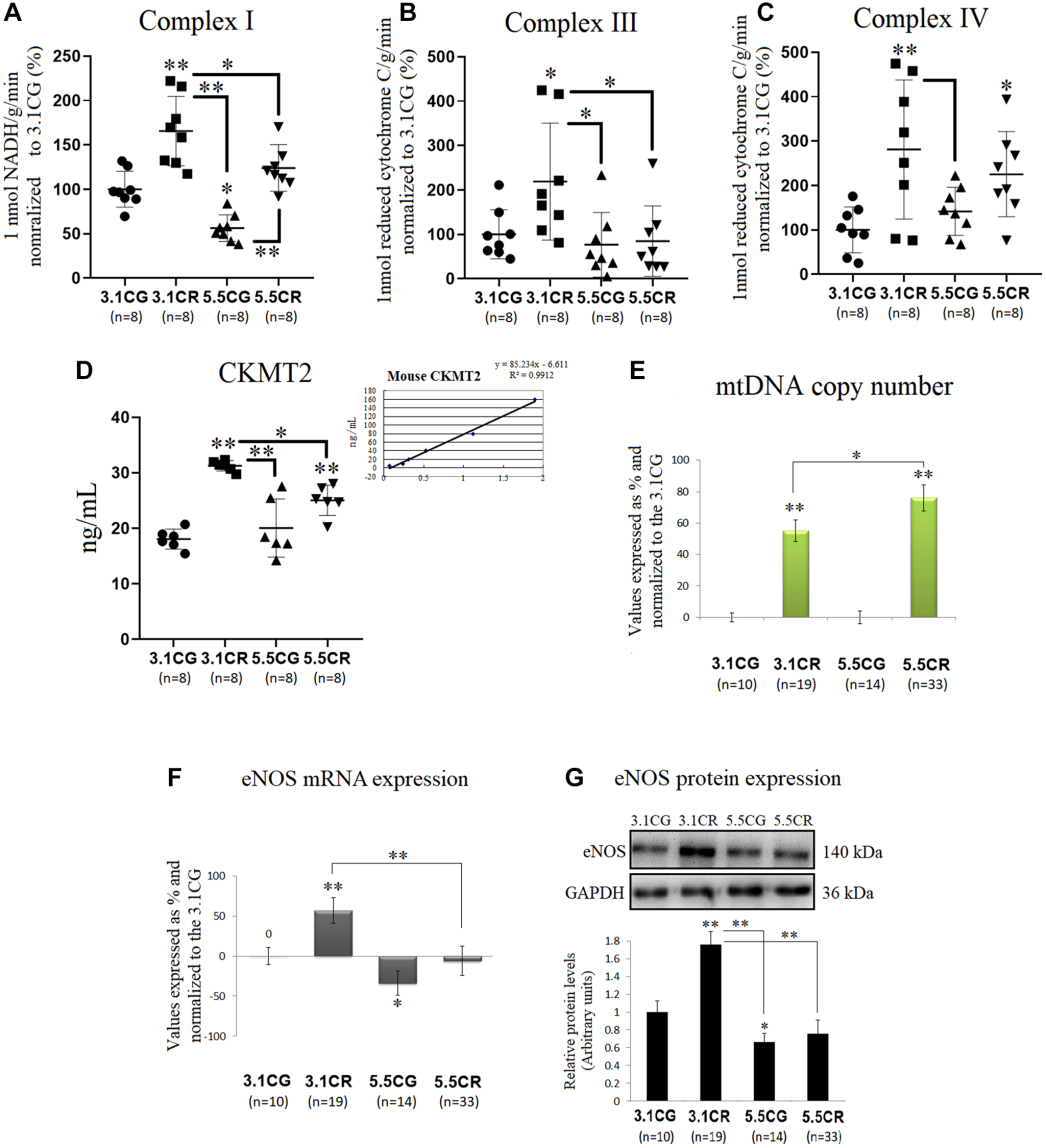
**Mitochondrial energetics, mtDNA copy numbers and eNOS expression.** OXPHOS activity in the WHT of mice was spectrophotometrically assessed through analysis of the activity of individual OXPHOS complexes I (**A**), II (**B**), and IV (**C**). Complex enzymatic activity was determined according to the consumption of 1 nmol of NADH (complex I) or reduced cytochrome C (complex III, IV) in 1 g of tissue/min in the reaction system. For each value expressed as %, the 3.1CG value was set to 100, and the compared samples were normalized to this level. The enzymatic activity of CKMT2 in WHT was analyzed with a Mouse CKMT2 ELISA Kit (**D**). The values were spectrophotometrically obtained and converted to ng/ml by normalization to standard values (**D**, lower-right graph). MtDNA copy numbers (**E**) were analyzed with a Detroit-Mouse-mt-DNA analysis kit. For each group, the value is expressed as a percentage (%); the 3.1CG value was set to 0, and the rest of the groups were normalized to this level for comparison. For eNOS (**F**) and 18S rRNA (reference gene) expression, each primer was analyzed with SYBR Green fluorescence detection, and the transcript levels of eNOS were normalized to those of the endogenous control 18S rRNA. (**G**) eNOS immunoblot results and relative protein values were obtained by Western blot analysis and quantified with Image Lab 6.1 software. The values were normalized to GAPDH expression; the 3.1CG expression was set as 1.0. The data are the mean ± SD. ^*^P < 0.05; ^**^P < 0.01 vs. the 3.1CG group, unless otherwise specified.

Mitochondrial energetics in the WHT of mice were spectrophotometrically assessed through the activity of individual OXPHOS complexes I (Figure 4A), II (Figure 4B), and IV (Figure 4C). Complex enzymatic activity was determined according to the consumption of 1 nmol of NADH (complex I) or reduced cytochrome C (complex III, IV) in 1 g of tissue/min in the reaction system.

CR in animals previously fed standard food (in the 3.1CR group), significantly stimulated the activity of mitochondrial complexes I, III, and IV (vs. those in the 3.1CG and 5.5CG groups). In contrast, CR in animals previously fed the HCD (in the 5.5CR group) only improved complex IV activity (vs. that in the 3.1CG group). Mice fed only the HCD (in the 5.5CG group) showed significant reductions in complex I activity (vs. the level in the 3.1CG group) without significant changes in complexes III and IV.

We also analyzed CKMT2, which is responsible for the transfer of high-energy phosphate from mitochondria to the cytosolic carrier creatine (Figure 4D). Our findings showed that CR markedly enhanced CKMT2 activity in both CR-treated groups (3.1CR and 5.5CR groups vs. the 3.1CG group). However, the CKMT2 levels in the 3.1CR group were significantly higher than those in the 5.5CR group.

MtDNA copy numbers were also analyzed, and we found increased levels in both groups subjected to CR (Figure 4E). These results are consistent with scientific data suggesting that CR induces mitochondrial proliferation in humans and rodents [52, 53]. However, the mtDNA copy number in the 5.5CR group (76% ± 8.3) was significantly higher than that in the 3.1CR group (55%, ± 7). To determine whether these findings correlate with mitochondrial biogenesis, we assayed the mRNA (Figure 4F) and protein (Figure 4G) expression of eNOS, which plays roles in the induction of mitochondrial biogenesis [53, 54]. We found that eNOS expression levels were upregulated in the 3.1CR group (vs. the 3.1CG group), while the 5.5CR group showed no significant changes. However, the expression in the 5.5CR group was significantly lower than that in the 3.1CR group. The interpretation of eNOS expression may suggest that the increase in mtDNA copy number in the 5.5CR group does not reflect an increase in mitochondrial biogenesis.

### Expression of the AMPK-SIRT1-mTOR-PGC-1α nutrient sensors

**Figure 5:**
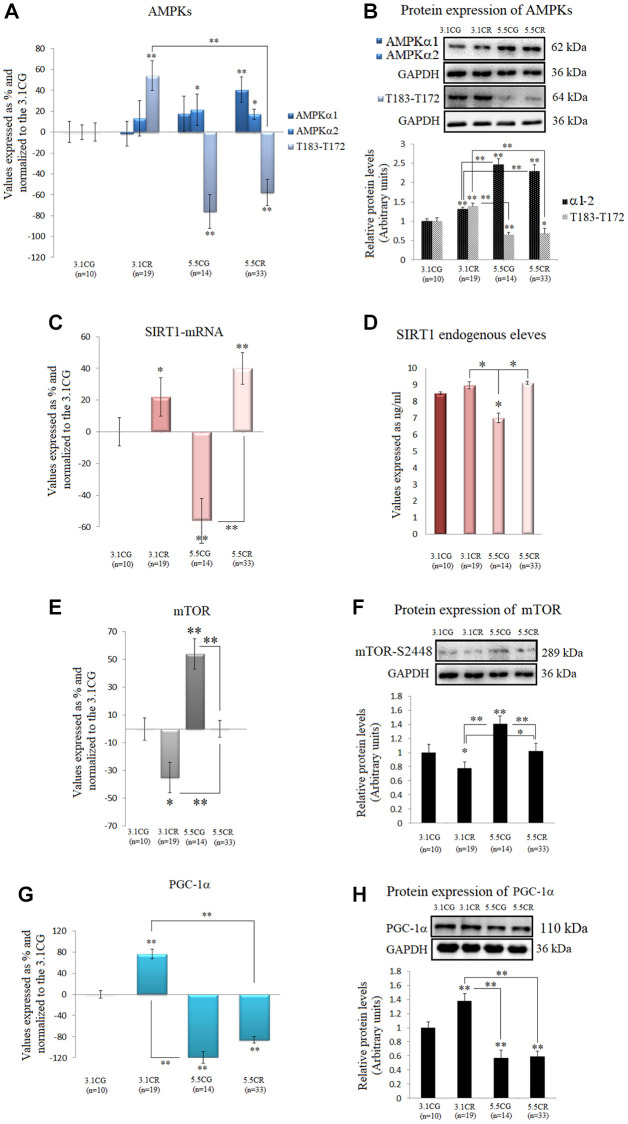
**Expression of the AMPK-SIRT1-mTOR-PGC-1α nutrient sensors.** The isoforms of the catalytic α-subunit of AMPK were analyzed by (**A**) qPCR (mRNA levels) and (**B**) Western blotting (protein levels^†^). T183-T172: AMPK α1 phosphorylated at T183 and AMPK α2 phosphorylated at T172. SIRT1 mRNA expression (**C**). SIRT1 endogenous levels (**D**). mTOR mRNA expression (**E**). Protein levels of mTOR phosphorylated at S2448^†^ (**F**). PGC-1α mRNA expression (**G**). PGC-1α protein levels (**H**). mRNA expression in the WHT was analyzed by qPCR with SYBR Green fluorescence detection, and the transcript levels of the target genes were normalized to those of the endogenous control 18S rRNA. For each group, the value is expressed as a percentage (%); the 3.1CG value was set to 0, and the values for the rest of the groups were normalized to this level for comparison. Immunoblot results and protein expression results were obtained by Western blot analysis and quantified with Image Lab 6.1 software. The values were normalized to GAPDH expression; the 3.1CG expression was set to 1.0. The data are the mean ± SD. ^*^P < 0.05; ^**^P < 0.01 vs. the 3.1CG group, unless otherwise specified. ^†^AMPKα1-α2 and mTOR-S2448 share the same GAPDH loading control.

We observed in this work that the mRNA and protein expression levels of the two isoforms of the catalytic α-subunit of AMPK were upregulated in the animals subjected to HCD feeding before CR (5.5CR animals) but downregulated in the 3.1CR animals (Figure 5A and 5B). Nonetheless, AMPK activity, as assessed by AMPK α1 phosphorylation at threonine 183 and AMPK α2 phosphorylation at threonine 172 (T183-T172), was significantly elevated in the 3.1CR group and downregulated in both groups fed the HCD (the 5.5CG and 5.5CR groups).

We also observed increases in SIRT1 mRNA levels (Figure 5C) in the animals subjected to CR (3.1CR and 5.5CR animals). However, the protein levels were not significantly altered (vs. those in the 3.1CG group; Figure 5D).

Furthermore, mTOR transcription and mTOR phosphorylation, whose inhibition is associated with mitochondrial biogenesis, were also assessed. Although there are four characterized phosphorylation sites in mTOR, we analyzed phosphorylation at serine 2448 (S2448) by S6K because the mTORC1 complex, which is known to confer cardioprotection, contains mainly mTOR phosphorylated at S2448 [55].

Our results showed downregulation of mTOR mRNA and S2448-phosphorylated mTOR protein in the 3.1CR group (vs. the 3.1CG group). However, the values in the 5.5CR group were higher than those in the 3.1CR group (Figure 5E and 5F).

Autophosphorylation at S2481 was also assessed by Western blot analysis, but none of the experimental groups or CGs showed phosphorylation activity at this site (data not shown).

We also analyzed PGC-1α because phosphorylation of AMPK and deacetylation of SIRT1 affect PGC-1α expression; thus, AMPK, SIRT1, and PGC-1α might act as a coordinated network to regulate metabolic fitness [26]. Consistent with our results regarding AMPK activity, PGC-1α mRNA and protein values were upregulated in the 3.1CR animals and markedly downregulated in the animals fed the HCD (Figure 5G and 5H).

### Telomerase activity and TERT mRNA levels as surrogates for telomerase enzyme activity

**Figure 6:**
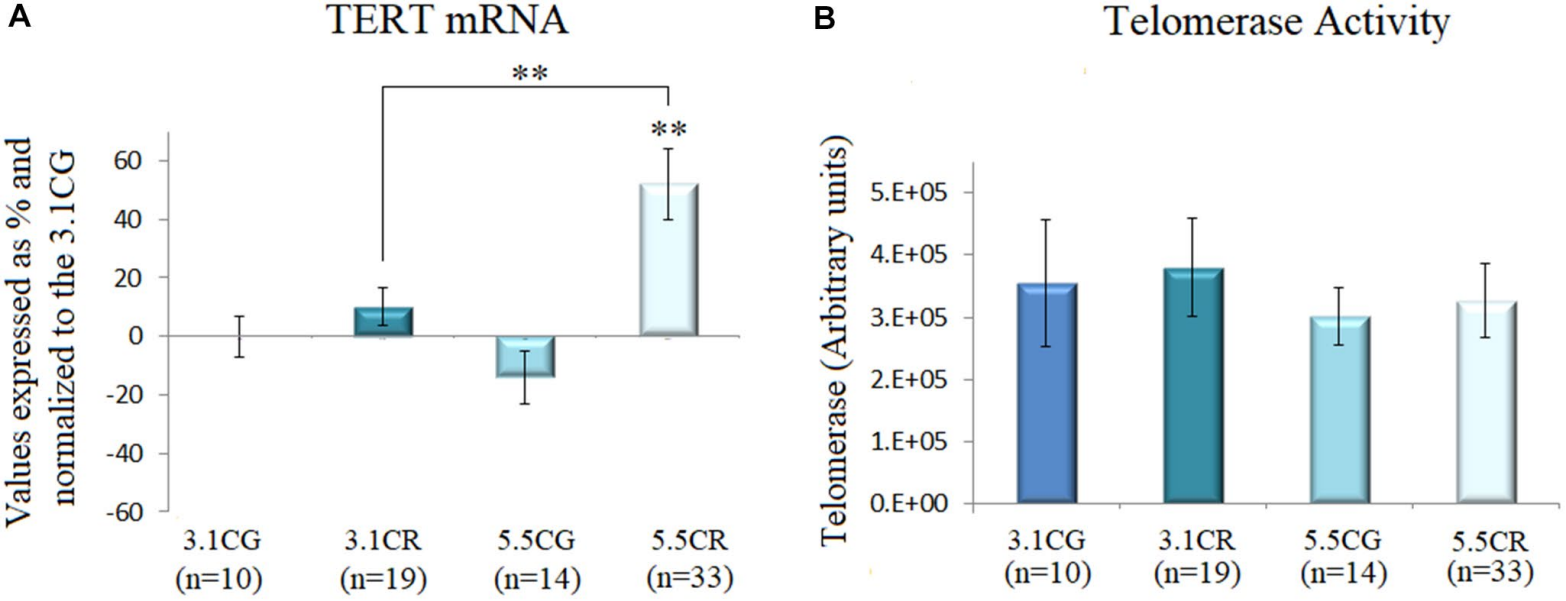
**Telomerase activity.** TERT mRNA expression (**A**). cDNA was obtained from WHT with a Takara RR047Q kit. qPCR was performed with 100 ng of target DNA. Significant differences in gene expression between groups are expressed as percentage (%) values. For each gene expressed as %, the 3.1CG value was set to 0, and the values in the compared samples were normalized to this level. Positive % values represent upregulation. Negative values represent downregulation. Each marker was analyzed with SYBR Green fluorescence detection, and the transcript levels of the markers were normalized to those of the endogenous control 18S rRNA. Telomerase activity (**B**) was assayed with a TRAPeze RT Telomerase Detection Kit (Millipore) for fluorometric detection and real-time quantification. The telomerase values are arbitrary units relative to TSR8 amplification, as specified in the manufacturer’s manual. The data are the mean ± SD. ^*^P < 0.05; ^**^P < 0.01 vs. the 3.1CG group.

We observed in this study that the groups subjected to CR (the 3.1CR and 5.5CR groups) showed upregulated TERT expression (Figure 6A). However, only the upregulation in the 5.5CR group was statistically significant (vs. the 3.1CG and 3.1CR groups; *p* < 0.01). Moreover, it is important to note that while TERT mRNA expression analysis is widely used as an indirect method for assessment of telomerase enzyme activity, there is now evidence that TERT mRNA is expressed in normal cells, so we interpreted the data in this work with caution [56].

Then, we performed fluorometric detection and real-time quantification of telomerase activity with a TRAPeze RT Telomerase Detection Kit (Millipore; Figure 6B). Although we observed slight changes in the telomerase units in the 3.1CR and 5.5CG groups, the results were not statistically significant.

### Expression of Adiponectin and Adiponectin receptors

**Figure 7:**
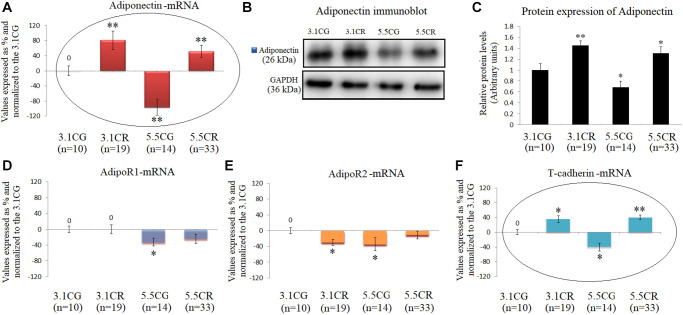
**Expression of Adiponectin and Adiponectin receptors.** mRNA levels of Adiponectin in WHT (**A**). Immunoblot results (**B**) and protein expression of Adiponectin (**C**). mRNA expression of the Adiponectin receptors AdipoR1 (**D**), AdipoR2 (**E**) and T-cadherin (**F**). The transcripts were analyzed by qPCR. Significant differences between groups are expressed as % values. For each gene expressed as %, the 3.1CG value was set to 0, and the values of the compared samples were normalized to this level. Positive values represent upregulation. Negative values represent downregulation. Each marker was analyzed with SYBR Green fluorescence detection, and the transcript levels of the target genes were normalized to those of the endogenous control 18S rRNA. Adiponectin protein expression was obtained by Western blot analysis and quantified with Image Lab 6.1 software. The values were normalized to GAPDH expression; the 3.1CG expression was set to 1.0. The data are the mean ± SD. ^*^P < 0.05; ^**^P < 0.01 vs. the 3.1CG group, unless otherwise specified.

Adiponectin in WHT was examined by qPCR (Figure 7A) and Western blot analyses (Figure 7B and 7C).

The mRNA and protein levels were significantly increased in the animals exposed to CR and decreased in the 5.5CG animals (vs. the 3.1CG animals). Moreover, the gene and protein expression of adiponectin in the experimental groups and CGs followed the same patterns as those of the adiponectin receptor T-cadherin (Figure 7E), while the receptors AdipoR1 and AdipoR2 presented no correlations with the expression of adiponectin (Figure 7D and 7E).

### Expression of CPC- and CSC-related markers

**Figure 8:**
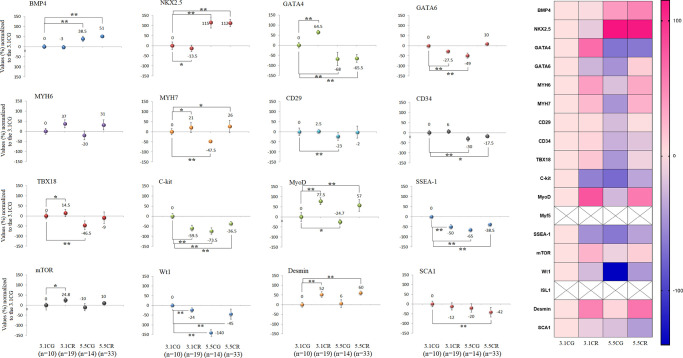
**Expression of CPC- and CSC-related markers.** cDNA was obtained from WHT with a Takara RR047Q kit. qPCR was conducted with 100 ng of target DNA. Significant differences in gene expression between groups are expressed as percentage (%) values. For each gene expressed as %, the 3.1CG value was set to 0, and the values of the compared samples were normalized to this level. Positive % values represent upregulation. Negative values represent downregulation. Each marker was analyzed with SYBR Green fluorescence detection, and the transcript levels of the markers were normalized to those of the endogenous control 18S rRNA. The data are the mean ± SD. ^*^P < 0.05; ^**^P < 0.01 vs. the 3.1CG group. A hierarchical clustering illustration for the up-/downregulation of genes analyzed from RT–PCR array data is shown on the right side of Figure 8. The right bar depicts the colors for upregulation and downregulation expressed as percentages.

Some of the most heavily analyzed cardiomyocyte markers, such as CD34, CD29, Connexin-37, Connexin-40, GATA4, GATA6, ISL1, MyoD, Myf5, MYH6, MYH7, SSEA-1, TBX18, Wt1, BMP4, C-KIT, Desmin, and NKX2.5, were analyzed in this work by qPCR in WHT from the different groups (Figure 8).

After 13 weeks of CR, the gene expression patterns differed between the animals exposed to CR with different alimentary backgrounds (3.1CR and 5.5CR animals). For instance, BMP4 and NKX2.5 were strongly upregulated (51%, ± 5; 112%, ± 18, respectively) in the 5.5CR group, while no significant change or downregulation was observed in the 3.1CR group (compared to the 3.1CG group). While GATA4, CD34, Connexin 37, and TBX18 were downregulated in the 5.5CR group (−65.5%, ± 20; −17.5%, 6; −30%, ± 13; −9%, ± 29, respectively), CD34 and Connexin 37 were not significantly altered in the 3.1CR group, and GATA4 and TBX18 were upregulated.

In addition, GATA6 was downregulated (to −27.5%, + 8) in the 3.1CR group, while it was slightly upregulated in the 5.5CR group (to 10%, + 7; vs. the level in the 3.1CG group).

Moreover, C-kit and SSEA-1, which are heavily studied cardiac markers linked with CSCs and CPCs, were significantly downregulated in the CR groups and the 5.5CG group (vs. the 3.1CG group). The same expression patterns were observed for Wt1.

## DISCUSSION

In this study, we compared the effects of CR on the WHT of mice previously fed standard food or an HCD.

In response to energy deficiency, the 3.1CR and 5.5CR displayed decreased body weights and heart weights, and improved insulin sensitivity, with no significant changes in cardiac fat content or fat infiltration. However, transcriptional and translational variability, which has morphological, functional, and metabolic implications, was evidenced in CR-treated animals with contrasting alimentary backgrounds.

For instance, the 3.1CR featured cardiac remodeling due to decreased IVS-d-s, LVID-d-s, and LVPW-d values, while EF and FS, were markedly improved. These and other geometric changes were accompanied by enhanced activity of the OXPHOS complexes I, III, and IV (responsible for generating mitochondrial membrane potential; ΔΨm), and CKMT2 enzymatic activity (a central component of the PCr/Cr-shuttle process, essential for cardiac cell homeostasis).

The 5.5CR showed changes in cardiac geometry only in terms of decreased IVS-d and IVS-s values. Most importantly, EF and FS values were significantly lower than in the 3.1CR. These animals presented improved OXPHOS complex IV and CKMT2 activity, indicating that the efficacy of CR was compromised in animals previously fed an HCD.

The 5.5CG were heavier, with higher cardiac fat content and lipid infiltration, than those that consumed the standard diet. Although the heart weights in the 5.5CG appeared elevated, they were not significantly different from those in the 3.1CG. Furthermore, the 5.5CG developed insulin resistance.

In terms of cardiac remodeling and function, the 5.5CG also featured increased IVS-d and IVS-s values, indicative of LV hypertrophy, and poor LV performance with muscle contractile dysfunction, estimated by EF. FS, which is dependent on LV preload, afterload, and contractility, was not affected by the HCD. These geometric and functional adaptations were accompanied by a reduction in OXPHOS complex I activity while complex III, IV, and CKMT2 activity levels remained unaltered. Nonetheless, as complex I is the primary entry point for electrons to the respiratory chain, decreased complex I activity could represent an alteration in the rate-limiting step of overall mitochondrial respiration and energy metabolism [57].

Notably, although the remarkable mechanical and functional adjustments were seen in the 3.1CR due to CR, CD-1 mice appeared to be slightly resistant to developing cardiac abnormalities when treated with a long-term HCD. Thus, we hypothesize that the above adaptations observed in the 5.5CG could be attributable to peculiarities of the strain.

Changes in cardiac energetics were also genetically assessed. CR promoted mitochondrial abundance, as indicated by increased amounts of mtDNA [58], in both CR-treated groups. However, abundance was not associated with biogenesis. This was confirmed by the upregulation of the mRNA and protein expression of eNOS, PGC1-α, AMPK activity, and dephosphorylation of mTOR in the 3.1CR, compared to the 5.5CR.

We also analyzed telomerase activity and TERT mRNA expression as a surrogate for telomerase enzyme activity [59–64]. Although we observed that TERT mRNA levels in the 5.5CR were upregulated, fluorometric quantification showed no significant differences between groups. Nevertheless, slight increases in telomerase units in the 3.1CR and a decrease in the 5.5CG were noticed, suggesting that a long-term CR analysis might be necessary to determine whether our findings are time-dependent.

Regarding AMPK-SIRT1-mTOR and –PGC-1α networks, CR increased AMPK activity in the 3.1CR, but comparable outcomes were not produced in the 5.5CR. Indeed, AMPK activity in the 5.5CR was significantly lower than that in the 3.1CG. AMPK activity has previously been reported to be reduced during the development of obesity, contributing to insulin resistance, metabolic syndrome, and related pathologies. Consistent with our findings, Gélinas, R. et al. (2018) demonstrated that submaximal AMPK activation appears to counteract cardiac hypertrophy by reducing O-GlcNAcylation [65]. AMPK modulates the activity of the downstream target PGC-1α, and the resulting reductions in the mRNA and protein expression of PGC-1α (in line with our results in the HCD groups) are indicators of cardiac dysfunction, compromised energy metabolism, and subsequent development of systolic heart failure [66]. In this work, AMPK phosphorylation was also associated with PGC-1α expression. In addition, both PGC-1α expression and AMPK activity followed similar activation patterns, with marked improvements in mitochondrial OXPHOS complex I, III, and IV activity in the 3.1CR and OXPHOS complex I impairment found only in the 5.5CG. Both energy-nutrient sensors appear to interact with several other activators of mitochondrial biogenesis, such as eNOS and adiponectin [67]. In addition, Zhu, X. et al. (2019) demonstrated that specific moderate overexpression of PGC-1α is sufficient to improve cardiac function [68], which is in line with our observations in the 3.1CR.

It has been documented that SIRT1 responds differently to specific stressors [69]. Although we observed transcriptional upregulation of SIRT1 in the CR-treated mice, the endogenous levels were not significantly elevated (vs. those in the 3.1CG), apparently due to the short-term CR implementation, as other studies have shown marked increases in SIRT1 expression after long-term CR [70, 71].

It is important to note that the regulatory properties of SIRT1 might nonetheless be remarkably intricate, as SIRT1 activity also relies on intracellular nicotinamide levels [72], posttranslational modifications [68], and interactions with several other protein-mediators [73, 74].

The mTOR analysis showed mTOR-S2448 mRNA and protein downregulation in the 3.1CR, vs. the CGs; interestingly, however, it also revealed downregulation in the 3.1CR vs. the 5.5CR. Moreover, mTOR expressions in the 5.5CR was not different from that in the 3.1CG. (Figure 5E and 5F). These results are in line with similar findings in the myocardium in male C57BL/6J mice [71]. In addition, our findings regarding mTOR expression and cardiac remodeling in the 5.5CG coincided, to some extent, with recent data indicating that mTORC1 activation via inducible cardiac-specific TSC2 knockdown results in hypertrophy without contractile dysfunction [75].

Due to the numerous antiaging properties of adiponectin and its receptors and the recognition of interactions of these molecules with a growing number of sensory molecules and regulatory networks of metabolic homeostasis, we analyzed the expression of these molecules. Adiponectin/AdipoR signaling is known to activate the PGC-1α–AMPK–SIRT1 pathway and positively regulate oxidative stress-detoxifying genes, thus alleviating oxidative stress in tissues [76]. Our results showed that the elevated transcript and protein levels of adiponectin (in the 3.1CR and 5.5CR) were not correlated with the levels of AdipoRs but were correlated with the mRNA level of t-cadherin (Figure 7A and 7F), one of the adiponectin receptors widely expressed in the heart and blood vessels [77].

It has been reported that adiponectin accumulates in the heart through interaction with T-cadherin [78], which in turn contributes to diminishing pathological cardiac remodeling, promoting revascularization, and exerting vasculo-protective actions, among other effects [78–83].

In our data analysis, upregulation of adiponectin/t-cadherin was associated with improved LV systolic performance and muscle contractility, enhanced cardiac mitochondrial respiration/energy production, improved AMPK activity, and eNOS and PGC-1α expression. We observed downregulation of adiponectin/t-cadherin in the 5.5CG that was, in turn, associated with cardiac hypertrophy, poor LV performance, and signs of impaired muscle contractility. Adiponectin/t-cadherin expression in the 5.5CG was also associated with diminished mitochondrial OXPHOS complex I activity, eNOS and PGC-1α transcriptional and translational expression, and AMPK dephosphorylation. Consistent with our results, a similar approach by Rui G. et al. (2013) has demonstrated that adiponectin deficiency in obese mice due to a high-calorie fat feeding produces cardiac hypertrophy, decreased AMPK activity, and glucose intolerance, among other effects [84]. Additionally, diminished AMPK signaling in the myocardium in adiponectin-deficient mice has been associated with enhanced concentric cardiac hypertrophy [85]. Furthermore, Nakajima, T. et al. (2019) have shown that diminished mitochondrial OXPHOS capacity in the epicardial adipose tissue (EAT) is closely linked to decreased concentration of adiponectin in the EAT and to the severity of coronary atherosclerosis [86], which is in line with the reduced OXPHOS complex I activity observed in the WHT of the 5.5CG.

Notably, serum adiponectin levels increase mainly due to secretion by central adipose tissue, but they do not necessarily correlate with the tissue-specific expression of adiponectin and receptors. However, this may vary in different organs [87].

Concerning the genetic profiling of CPCs and CSCs in the WHT, the approach was not intended to reveal alterations in these cell pools explicitly. Some of the proposed transcripts are not specific to putative CPCs or CSCs, as they are also expressed in cardiomyocytes and fibroblasts. [88–91]. Furthermore, resident CPCs show mixed and overlapping expression of SC markers [92]. Hence, here, we present only evidence of how an HCD alters genetic patterns in the WHT of animals subjected to CR with the same BMI.

Interestingly, the level of BMP4, a member of the bone morphogenetic protein family and a mediator of cardiac hypertrophy [93, 94], was elevated in the 5.5CG (consistent with the increased IVS-d and IVS-s values in the 5.5CG) but also in the 5.5CR, which did not feature cardiac hypertrophy. Unlike BMP4, GATA4, another transcription factor related to cardiac hypertrophy, was downregulated in both groups fed an HCD. This may have been one reason why the CD-1 mice fed only with an HCD appeared to be slightly resistant to developing severe hypertrophy and cardiac remodeling. However, as growing evidence suggests that GATA4 regulates the transcriptional responses of other hypertrophy marker genes such as ANF, BNP, β-MHC, and Acta1 [95], and exhibits synergistic activity with NKX2.5 (HCD-treated mice exhibited antagonistic activity), further research is needed to understand the transcriptional downregulation of GATA4 in the animals fed an HCD.

To the best of our knowledge, how the expression of markers specific for CPCs or CSCs is impacted by HCD consumption in combination with CR has been overlooked, and much remains to be determined regarding the functions of transcription factors and how transcription factors are affected by dietary interventions.

We acknowledge that the limitations of this work exponentially escalate with the magnitude of the presented data and content being discussed. Thus, our results may not cover all aspects that affect or interact with the subject of study. Other aspects such as comparisons of serum with WHT to demonstrate alterations in proteomic profiles in murine models and analyses of adiponectin and insulin blood concentrations, NAD+, other major components of the PCr/Cr-shuttle process, and inflammatory cytokine levels would contribute to reinforcing the findings listed in this work.

Additionally, due to the number and characteristics of the experimental assays and the low amounts of mouse heart tissues, we were obligated to develop three separate cohorts of experimental animals. Although all mice in the different groups met the characterization requirements for the experimental animal model, we cannot rule out that some differences due to animal or cohort peculiarities may have existed.

One pitfall of our analysis of cardiac function was that it included EF and FS (used in this work as indicators of overall LV performance and muscle contractility) as well as LVmass and LVvolumes, which depend on geometrical assumptions of the LV (the left ventricle is assumed to be a sphere rather than an ovoid) obtained with M-mode echocardiography in the PLAX view.

In addition, because our experimental procedure was based on short-term CR, some of our data seemed to be time-dependent. In other words, a three-month period of CR was too short to demonstrate rescue in some categories of our observations. Thus, determining the adequate length of the dietary interventions and conducting a mechanistic rescue of the PGC-1α-AMPK-mTOR-related axes, which were negatively affected in the 5.5CR, will be prerequisites for future elucidation of our preliminary findings.

## CONCLUSIONS

Although our data exemplify the potential cardioprotective properties of short-term CR, we report here that past habits of caloric overload rerouted the effects of CR observed in the WHT of animals with a history of standard diet consumption.

Inconsistencies in the expression of mitochondrial-biogenesis-related markers and in regulatory networks, particularly the disruption of PGC-1α–AMPK-mTOR-related axes, were observed in CR-treated mice previously fed an HCD. In addition, differences in the activation patterns of specific markers of CPC and CSC populations, corroborated the existence of transient and perhaps irreversible disruptions at the genetic level, with potential morphological, metabolic, and functional implications.

Identifying optimal caloric-dietary strategies by circumventing the risk factors associated with nutritional inadequacy from lack of macro/micronutrients to accomplish long-term anti-aging effects will undoubtedly require further exploration, in combination with larger data sets from other tissues and organs at higher levels of organization within the organ system.

Overcoming these challenges will eventually increase our understanding and support the development of preventative and therapeutic strategies to promote health and longevity.

## Supplementary Materials

Supplementary Tables 1 and 3

Supplementary Table 2
