# Supplementary material for: Effects of caloric overload before caloric restriction in the murine heart
Source: Aging (Albany NY). 2022 Mar 28;14(6):2695–719. doi: 10.18632/aging.203967 (PMC9004582; doi:10.18632/aging.203967)
Supplement: Supplementary Tables 1 and 3 [file aging-14-203967-s001.pdf]

SUPPLEMENTARY TABLES

Supplementary Table 1. Standard food composition.

Number	Inspection item	Test result (%)	Test method
1	Crude protein	19.7	GB/T6432-1994 7.2
2	Coarse fiber	3	GB/T6434-2006
3	Coarse ash	5.94	GB/T6438-2007
4	Calcium	1.17	GB/T6436-2018
5	Total phosphorus	0.89	GB/T6437-2002
6	Crude fat	5.3	GB/T6433-2006 9.3
7	Water	9.5	GB/T6435-2014 8.1

Standard food composition supplied by the manufacturer company: Beijing Keao Xieli Feed Co., Ltd. (Beijing Chaoyang district, Yangshan road, number 4). [*]The standard food provides 3.1 kcal/kg of energy.

Supplementary Table 3. List of primers.

Oligo name	Sequence Forward (5′ to 3′)	Sequence Reverse (5′ to 3′)	Product Size (bp)	Accession Number[a]
18sRNA	GTAACCCTTTGAACCCCATT	CCATCCAATCGGTAGTAGCG		
Adiponectin	AAGGACAAGGCCGTTCTCT	TATGGGTAGTTGCAGTCAGTTGG	219	NM_009605.5
Adipo R1	TTCTTCCTCATGGCTGTGATGT	AAGAAGCGCTCAGGAATTCG	71	XM_021197931.2
AdipoR2	TGCAGCCATTATAGTCTCCCAG	GAATGATTCCACTCAGGCCTAG	101	XM_021190928.2
ATP5C1	CATGGACAACGCCAGCAAGA	TTTACCTCTTGTCTGAGGATGCAAC	165	NM_001112738.1
BMP4	CTGCCTGATCTCAGCGGCACCCACATC	GCCGGAGGGCCAAGCGTAGCCCTAAG	378	XM_029468692.1
CD29	AATGGAGTGAATGGGACAGG	TCTGTGAAGCCCAGAGGTTT	149	XM_029532371.1
CD34	GTTATTTCCTGATGAACCGTCG	CTCCACCATTCTCCGTGTAATA	84	XM_021198209.1
C-kit	GCTTTTCTTACCAGGTGCCAAA	GAGGATATTTCTGGCTGCCAAGT	218	XM_017320687.2
Connexin-37	CAACCTGACCACAGAGGAGAG	CTTAGAAGCAGAGCTGCTGG	112	XM_021160828.1
Connexin-40	CAGCCTGGCTGAACTCTACCA	CTGCCGTGACTTGCCAAAG	67	XM_021196653.2
Desmin	GATGAGGCAGATGAGGGAG	TGAGAGCAGAGAAGGTCTGG	245	XM_021199367.2
eNOS	CGAAGCGTGTGAAGGCAAC	TTGTACGGGCCTGACATTTCC	250	XM_006535639.4
eNOS	TGTGACCCTCACCGCTACAA	GCACAATCCAGGCCCAATC	247	XM_006535639.4
GAPDH	AGGTCGGTGTGAACGGATTTG	GGGGTCGTTGATGGCAACA	95	XM_017321385.2
GATA4	GCCGAGGGAGCCGCCTACAC	TGGGGTGTCTTCCAGGGTTGG	328	XM_011244957.3
GATA6	AGGAGATGTACCAGACCC	TGCCGTATGGAGGGCTGT	273	XM_021150661.2
ISL-1	CGCGTGCGGACTGTGCTGAAC	TTGGGCTGCTGCTGCTGGAGT	209	XM_029544024.1
mAMPKα1	GTCGACGTAGCTCCAAGACC	ATCGTTTTCCAGTCCCTGTG	232	XM_011245321.3
mAMPKα2	CGCCTCTAGTCCTCCATCAG	ATGTCACACGCTTTGCTCTG	219	XM_029539581.1
mTERT	TCTACCGCACTTTGGTTGCC	CAGCACGTTTCTCTCGTTGC	155	NM_001362388.1
mTOR	TTCAATCCATAGCCCCGTCT	CAAAGAGCTGCATCACTCGT	150	XM_006539077.3
Myf5	CTGTCTGGTCCCGAAAGAAC	AAGCAATCCAAGCTGGACAC	103	XM_006513319.2
MYH6	ACATGAAGGAGGAGTTTGGG	GCACTTGGAGCTGTAGGTCA	123	XM_006518678.3
MYH7	AAGGGCCTGAATGAGGAGTAGATC	TGCAAAGGCTCCAGGTCTGA	80	XM_021181495.2
MyoD	CCCCGGCGGCAGAATGGCTACG	GGTCTGGGTTCCCTGTTCTGTGT	234	NM_010866.2
NDUFS3	GCTTCGAGGGACATCCTTTC	AGTTACTTGGTTTCAGGCTTCT	223	XM_021152446.2
NKX2.5	CGACGGAAGCCACGCGTGCT	CCGCTGTCGCTTGCACTTG	180	XM_006523797.4
PGC-1α	TATGGAGTGACATAGAGTGTGCT	CCACTTCAATCCACCCAGAAAG	134	XM_036164894.1
SCA-1	ATGGAGAACAACAAAACCTCAGT	TTGCTCCCATGTATGGTCTTTAC	74	XM_030243266.1
SSEA-1	CTTTGTGCCTTATGGCTACC	TTGGCTCAGTTGGTGGTAGT	160	XM_021207694.2
TBX18	GTGGAGTCATACGCATTCTGGA	GTGAGGATGTGTAGCAGGGACA	141	NM_023814.4
T-cadherin	CATCGAAGCTCAAGATATGG	GATTTCCATTGATGATGGTG	230	XM_021169677.2
TIMM9	AATATGGCTGCACAGATACC	TTCAGGTTTCACCTCTCTTG	135	XM_017315088.2
TOMM20	GAAACAGAAGCTTGCTAAGGAG	GTCACCTTGTGCTAGTAACTCT	127	XM_021170680.2
TOMM40	TGAACAGTAACTGGATCGTG	GGAGGACATCAAGTCTTTCC	390	XM_030242769.1
Wt1	CAGATGAACCTAGGAGCTACCTTAAA	TGCCCTTCTGTCCATTTCA	74	XM_029537108.1
